# Urinary Proteomics Pilot Study for Biomarker Discovery and Diagnosis in Heart Failure with Reduced Ejection Fraction

**DOI:** 10.1371/journal.pone.0157167

**Published:** 2016-06-16

**Authors:** Kasper Rossing, Helle Skovmand Bosselmann, Finn Gustafsson, Zhen-Yu Zhang, Yu-Mei Gu, Tatiana Kuznetsova, Esther Nkuipou-Kenfack, Harald Mischak, Jan A. Staessen, Thomas Koeck, Morten Schou

**Affiliations:** 1 Department of Cardiology, Rigshospitalet, University Hospital of Copenhagen, Copenhagen, Denmark; 2 Department of Cardio-, Nephro-, and Endocrinology, North Zealand Hospital, University of Copenhagen, Copenhagen, Denmark; 3 Studies Coordinating Centre, Research Unit Hypertension and Cardiovascular Epidemiology, KU Leuven Department of Cardiovascular Sciences, University of Leuven, Leuven, Belgium; 4 Mosaiques Diagnostics and Therapeutics AG, Hanover, Germany; 5 Institute of Cardiovascular and Medical Sciences, University of Glasgow, Glasgow, United Kingdom; 6 Institute for Clinical Medicine, Herlev Hospital, Herlev, Denmark; Providence VA Medical Center and Brown University, UNITED STATES

## Abstract

**Background:**

Biomarker discovery and new insights into the pathophysiology of heart failure with reduced ejection fraction (HFrEF) may emerge from recent advances in high-throughput urinary proteomics. This could lead to improved diagnosis, risk stratification and management of HFrEF.

**Methods and Results:**

Urine samples were analyzed by on-line capillary electrophoresis coupled to electrospray ionization micro time-of-flight mass spectrometry (CE-MS) to generate individual urinary proteome profiles. In an initial biomarker discovery cohort, analysis of urinary proteome profiles from 33 HFrEF patients and 29 age- and sex-matched individuals without HFrEF resulted in identification of 103 peptides that were significantly differentially excreted in HFrEF. These 103 peptides were used to establish the support vector machine-based HFrEF classifier HFrEF103. In a subsequent validation cohort, HFrEF103 very accurately (area under the curve, AUC = 0.972) discriminated between HFrEF patients (N = 94, sensitivity = 93.6%) and control individuals with and without impaired renal function and hypertension (N = 552, specificity = 92.9%). Interestingly, HFrEF103 showed low sensitivity (12.6%) in individuals with diastolic left ventricular dysfunction (N = 176). The HFrEF-related peptide biomarkers mainly included fragments of fibrillar type I and III collagen but also, e.g., of fibrinogen beta and alpha-1-antitrypsin.

**Conclusion:**

CE-MS based urine proteome analysis served as a sensitive tool to determine a vast array of HFrEF-related urinary peptide biomarkers which might help improving our understanding and diagnosis of heart failure.

## Introduction

Heart failure is a complex clinical syndrome characterized by impaired ventricular filling and/or ejection of blood resulting in the disability of the heart to pump a sufficient amount of blood to meet the metabolic demands of the body. Heart failure with reduced ejection fraction (HFrEF; left ventricular ejection fraction < 45%) is a potential end-stage of various cardiac diseases and represents an enormous public health and socioeconomic burden [1]. Different aetiologies may lead to the HFrEF phenotype including myocardial ischemia, hypertension, diabetes, valvular heart disease, arrhythmias and inherited cardiomyopathy. However, in the clinical setting it is often difficult to clearly identify all contributing factors. Many of the currently used biomarkers only depict part of the pathology [2]. Diagnosis, prognostication and follow-up of HFrEF patients based on currently utilized clinical, laboratory and imaging markers in the everyday practice is therefore often complex [3,4]. A new multi-biomarker-based HFrEF classifier that identifies distinct HFrEF-related molecular phenotypic expressions may provide additional (differential) diagnostic and prognostic value and prove beneficial in guiding therapy and identify new targets of treatment. It may especially help to identify and stratify asymptomatic individuals at an early stage of cardiac structural impairment.

The clinical use of proteomic analysis of body fluids like blood and urine is an emerging and promising field of research made possible through recent advances in high-throughput methods. As a non-hypothesis-driven approach, the identification of protein/peptide biomarkers by proteomic analysis may provide a novel modality for diagnosis, prognostication, and treatment guidance as well as for development of new treatment strategies [5]. Previous studies have used urine proteome analysis (UPA) to identify patterns of urinary peptide biomarkers for coronary artery disease and preclinical left ventricular diastolic dysfunction (LVDD) [5,6]. These biomarkers were utilized to establish specific disease classifiers. This approach has not yet been applied to HFrEF. Potential benefits of proteomic analysis for HFrEF management has been shown by Lemesle et al. who demonstrated that plasma multimarker proteomic profiling can predict cardiovascular mortality in patients with chronic heart failure [7].

The aim of the present case-control study was therefore to assess the feasibility of UPA for the identification of a HFrEF-related urinary peptide biomarker pattern and the usability of such a pattern to establish a diagnostic HFrEF classifying algorithm.

## Methods

### Study population

HFrEF patients were enrolled prospectively at their first visit to a heart failure clinic at the North Zealand Hospital in Denmark (N = 149) as described in detail previously [8]. Urine samples from these 149 HFrEF patients were analyzed by CE-MS-based UPA performed by Mosaiques Diagnostics GmbH (Hanover, Germany) and 127 passed all quality control criteria [9] and were thus included in the present study. All patients were known to have heart failure (HF) with left ventricular ejection fraction (LVEF) <45% [10] and were referred to the clinic for up-titration of guideline recommended therapy. To be included, the patients had to be in a stable condition with no hospital admissions for a minimum of 60 days and plasma creatinine had to be stable (+/- 10 μg/l) for a period of 60 days. Descriptions of coronary angiography were retrieved when available, for categorizing the patients as having non-ischemic or ischemic heart disease [11]. Patients collected a 24-hour urine sample, starting on the day before the exam and delivered spontaneously voided urine on the day of the exam for UPA. Fasting venous blood samples were taken and patients underwent echocardiography.

CE-MS based urinary proteome profiles of 581 control urine samples of individuals without heart failure and 176 urine samples of asymptomatic individuals diagnosed with preclinical LVDD [6] were provided by Mosaiques Diagnostics GmbH and originated from the Flemish Study on Environment, Genes and Health Outcomes (FLEMENGHO). Briefly, in this cohort left ventricular function was assessed by echocardiography and preclinical LVDD defined as (1) an abnormally low age-specific transmitral E/A ratio indicative of impaired relaxation, but without evidence of increased LV filling pressures (E/e' ≤8.5), (2) mildly-to-moderately elevated LV filling pressure (E/e' >8.5) and an E/A ratio within the normal age-specific range or (3) an elevated E/e' ratio and an abnormally low age-specific E/A ratio (combined dysfunction). Differences in durations between the transmitral A flow and the reverse PV flow during atrial systole (Ad < ARd + 10) and/or LA volume index (≥28 mL/m^2^) were checked to confirm possible elevation of the LV filling pressures in group 2. For staging LV diastolic dysfunction, the mitral inflow and TDI velocities were combined.

The study complies with the Declaration of Helsinki, and all subjects provided informed oral and written consent. The study was approved by the local Ethical Committee of the capital region of Denmark (H-1-2010-074) and Commissie Medische Ethiek van de Universitaire Ziekenhuizen Kuleuven, U.Z. Gasthuisberg E330 Leuven, Belgium (ML4804).

To identify and validate the HFrEF-related urinary peptide biomarkers potentially discriminating between HFrEF and healthy individuals, these HFrEF patients and healthy control individuals were divided into a biomarker discovery cohort and a validation cohort. Overall, study participants had rather well preserved kidney function (Table 1) but 38 HFrEF patients (29.9%) and 19 controls (3.4%) had moderate to severe chronic kidney disease (CKD) with an estimated glomerular filtration rate (eGFR) < 60 ml/min/1,73m^2^ (CKD stage 3–5).

**Table 1:** Demographics and clinical features of study participants.

	control (N = 581)	HFrEF (N = 127)	LVDD (N = 176)
Gender, male / female	268 / 283	95 / 32	77/99
Age, years	47 ± 13	70 ± 10*	64 ± 13*
NYHA I / II / III / IV	n.a.	28 / 62 / 34 / 3	n.a.
LVEF (%)	69 ± 6	32 ± 9*	70 ± 8
Aetiology (N; ischemic/non-ischemic)	n.a.	65/47	n.a.
Atrial fibrillation	n.a.	46	n.a.
Hypertension (N, (% of N_total_))	185 (32)	77 (61)	132 (75)
Systolic blood pressure (mm Hg)	126 ± 15	129 ± 22	140 ± 19*
Diastolic blood pressure (mm Hg)	80 ± 9	76 ± 13*	82 ± 10*
BMI (kg/m^2^)	26 ± 4	27 ± 6*	28 ± 5*
eGFR (MDRD; ml/min/1,73m^2^)	82 ± 15	72 ± 24*	72 ± 15*
Diabetes type 2 (N)	4	26	5

LVDD, left ventricular diastolic dysfunction; NYHA, New York Heart Association; LVEF, left ventricular ejection fraction; eGFR, estimated glomerular filtration rate

* One-way ANOVA in regard to control with P < 0.05

#### Selection of HFrEF patients and controls for biomarker discovery

For the discovery of HFrEF-related urinary peptide biomarkers, HFrEF patients have been selected to be representative of the patient cohort with regard to New York Heart Association (NYHA) class, left ventricular ejection fraction and ischemic and non-ischemic aetiology of HFrEF. However, with regard to kidney function the selection was only partly representative since patients with severely impaired kidney function (CKD stage 4 and 5; eGFR ≤ 30 ml/min/1.73m^2^) have not been considered for biomarker discovery to limit a bias in the HFrEF-relevant peptide biomarker pattern due to CKD-relevant peptides. HFrEF patients with cancer have also been excluded from biomarker discovery. Due to the fact that CKD is a common comorbidity in acute and/or chronic heart failure result in increased complications and mortality [12,13], HFrEF patients with an eGFR between 30 and 60 ml/min/1.73m^2^ were randomly selected in a number representative of the patient cohort. This resulted in the selection of 33 HFrEF patients for biomarker discovery comprising 13 patients with non-ischemic and 20 patients with ischemic aetiology. The controls were individuals from the FLEMENGHO cohort without cardiovascular conditions at baseline and/or during follow-up that were best matched with the HFrEF patients for age, sex and eGFR. The controls selected for biomarker discovery were thus only partly representative of the FLEMENGHO cohort. Individuals omitted in biomarker discovery were assessed in validation. The clinical characteristics of these selected patients and controls are presented in Table 2.

**Table 2:** Demographics and clinical features of individuals in the cohort for biomarker discovery and creation of the HFrEF classifiers.

	control (N = 29)	HFrEF-NI (N = 13)	HFrEF-I (N = 20)
Gender, male / female/ % female	21 / 8 / 27.6	11 / 2 / 15.4	15 / 5 / 25
Age, years (range)	67 ± 7 (49–79)	65 ± 8 (49–78)	72 ± 5* (64–81)
NYHA I / II / III / IV	n.a.	4 / 7 / 2 / 0	4 / 8 / 6 / 2
LVEF, %	71 ± 8	38 ± 8*	29 ± 8*
Atrial fibrillation	n.a.	7	3
Hypertension	20	0	0
Systolic blood pressure (mm Hg)	138 ± 14	123 ± 20*	120 ± 21*
Diastolic blood pressure (mm Hg)	80 ± 8	74 ± 14	74 ± 10*
BMI (kg/m^2^)	29 ± 6	28 ± 5	25 ± 4*
eGFR (MDRD; ml/min/1,73m^2^)	76 ± 12	79 ± 17	69 ± 27

NYHA, New York Heart Association; LVEF, left ventricular ejection fraction; eGFR, estimated glomerular filtration rate; HFrEF-NI, heart failure with reduced ejection fraction with non-ischemic etiology; HFrEF-I, heart failure with reduced ejection fraction with ischemic etiology

* One-way ANOVA in regard to control with P < 0.05

### Sample preparation and CE-MS analysis

All urine samples for CE-MS analyses were taken from spontaneously voided urine at the day of the exam and stored at -80°C until analysis. For proteomic analysis, a 0.7 mL aliquot of urine was thawed immediately before use and diluted with 0.7 mL of 2 M urea, 10 mM NH_4_OH containing 0.02% SDS. To remove higher molecular mass proteins, such as albumin and immunoglobulin G, the sample was ultra-filtered using Centrisart ultracentrifugation filter devices (20 kDa MWCO; Sartorius, Goettingen, Germany) at 3,000 rcf until 1.1 ml of filtrate was obtained. This filtrate was then applied onto a PD-10 desalting column (GE Healthcare, Uppsala, Sweden) equilibrated in 0.01% NH_4_OH in HPLC-grade in H_2_O (Roth, Germany) to decrease matrix effects by removing urea, electrolytes, salts, and to enrich polypeptides present. Finally, all samples were lyophilized, stored at 4°C, and suspended in HPLC-grade H_2_O shortly before CE-MS analyses, as described [14].

CE-MS analyses were performed using a P/ACE MDQ capillary electrophoresis system (Beckman Coulter, Fullerton, USA) on-line coupled to a micrOTOF MS (Bruker Daltonics, Bremen, Germany) as described previously [14,15]. The ESI sprayer (Agilent Technologies, Palo Alto, CA, USA) was grounded, and the ion spray interface potential was set between –4 and –4.5 kV. Data acquisition and MS acquisition methods were automatically controlled by the CE *via* contact-close-relays. Spectra were accumulated every 3 s, over a range of *m/z* 350 to 3000. Accuracy, precision, selectivity, sensitivity, reproducibility, and stability of the CE-MS measurements were demonstrated elsewhere [14].

#### Mass spectrometry data processing

Mass spectral peaks representing identical molecules at different charge states were deconvoluted into single masses using MosaiquesVisu software [16]. Only signals with z>1 observed in a minimum of 3 consecutive spectra with a signal-to-noise ratio of at least 4 were considered. Reference signals of 1770 urinary polypeptides were used for CE-time calibration by locally weighted regression. For normalization of analytical and urine dilution variances, signal intensities were normalized relative to 29 ‘‘housekeeping” peptides [17,18]. The obtained peak lists characterize each polypeptide by its molecular mass (Dalton; Da), normalized CE migration time (minutes; min) and normalized signal intensity. All detected peptides were deposited, matched, and annotated in a Microsoft SQL database allowing further statistical analysis [19]. For clustering, peptides in different samples were considered identical if mass deviation was <50 ppm. CE migration time was controlled to be below 0.35 minutes after calibration.

#### Sequencing of polypeptides

Identified heart failure biomarkers were *in silico* assigned to the previously sequenced peptides from Human urinary proteome database, version 2.0. Peptides from the Human urinary proteome database were sequenced as described elsewhere [20,21]. Briefly, urinary peptides were fragmented using different tandem mass spectrometry techniques with prior separation step with CE or HPLC. Fragmentation spectra were matched to the protein sequences from the up-to-date databases (IPI, NCBI nr and Uniprot) using MS/MS search engines MASCOT (Matrix Sciences Ltd., London, UK) and OMSSA (The National Center for Biotechnology Information, Bethesda, USA). Characteristic for urinary proteins post-translational modifications (PTM), such as hydroxylation of lysine and proline, and mass spectrometer specific settings were used. Identified peptide sequences from LC-MS analyses were verified by the comparison of experimental and theoretical CE migration time, which is dependent on the number of basic and neutral polar amino acid.

#### Definition of biomarkers

For biomarker discovery, statistical analysis of the selected urinary proteome profiles was performed using non-parametric Wilcoxon rank sum test. Only biomarkers that were found at a 70% frequency or higher in either case or control group were examined. The false discovery rate adjustments of Benjamini-Hochberg [22] were employed to correct for multiple testing. A p-value less than 0.05 was considered to be statistically significant.

#### Support vector machine (SVM) modelling

HFrEF-related peptide biomarkers were combined into single summary multidimensional classifying variables, hereinafter referred to as classifiers, based on non-ischemic and ischemic aetiologies, using the support-vector machine based MosaCluster proprietary software, version 1.7.0 [23]. These classifiers based on SVM modelling allowed the classification of samples in the high dimensional data space. MosaCluster calculated classification scores based on the amplitudes of the HFrEF biomarkers. Classification is performed by determining the Euclidian distance (defined as the SVM classification score) of the vector to a maximal margin hyperplane. The SVM classifier uses the log transformed intensities of x features (peptides) as coordinates in a x-dimensional space. It then builds a x-1 dimensional hyperplane that spans this space by performing a quadratic programming optimisation of a Lagrangian using the training labels only while allowing for samples to lie on the wrong side of the plane. For such mistakes in classification the SVM introduces a cost parameter C. Because non-separable problems in low dimensions may be separable in higher dimensions the SVM uses the Kernel-trick to transform the samples to a higher dimensional space. MosaCluster uses the standard radial basis functions as kernel. These functions are just Gaussians with the parameter gamma controlling their width. The optimal parameters C and gamma are found via e.g. leave one out cross validation error estimation. There are generally implemented in SVMs in all popular data mining software, particularly the kernlab cran contributed R package is a versatile tool for building SVM based-classifiers [24]. After identification of significant biomarkers and generation of different classifiers, they were assessed in a test set to check their performance.

### Other Measurements

Echocardiography was performed using a Vivid 9E [8,25] and Vivid7 Pro [6] (General Electric, Horten, Norway), and images were transferred to a remote workstation for offline analysis (Echopac, General Electric, Horten, Norway). Two dimensional parasternal images were used to determine LV dimensions and LVEF was determined from the biplane Simpson model. Blood pressure was the average of five consecutive auscultator readings obtained according to European guideline with a standard mercury sphygmomanometer with the participant in the seated position for at least 10 minutes. As described elsewhere, we applied a stringent quality control program to the blood pressure measurements, looking for digit and number preference [26,27]. Hypertension was defined as elevated blood pressure of at least 140 mm Hg systolic or 90 mm Hg diastolic at the time of inclusion in the study, use of antihypertensive drugs at the time of inclusion in the study and/or a history of elevated blood pressure. Body mass index was weight in kilograms divided by the square of height in meters. Glomerular filtration rate was estimated from the Chronic Kidney Disease Epidemiology Collaboration equation [28].

### Statistical methods and sample classification

By maximizing Youden’s index, we determined optimal thresholds for the HFrEF classifiers to differentiate normal individuals from HFrEF patients based on exact binomial calculations and were carried out in MedCalc version 12.7.3.0 (MedCalc Software, Mariakerke, Belgium, http://www.medcalc.be). Estimates of sensitivity and specificity and their confidence intervals (95% CI) were calculated based on tabulating the number of correctly classified samples and exact binomial calculations. The Receiver Operating Characteristic (ROC) plot was obtained and the area under the ROC curve (AUC) was evaluated. The reported unadjusted p-values were calculated using the natural logarithm-transformed intensities and the Gaussian approximation to the t-distribution. Statistical adjustment due to the existence of multiple test sets was performed by using the Westfall and Young maxT-procedure [29], by adjusting according to Bonferroni [30], and by applying the Benjamini-Hochberg function to the entire dataset (case vs. control) [31,32].

Means were compared using the large-sample z-test or ANOVA and proportions by Fisher’s exact test. Statistical significance was a 1-sided significance level of 0.05.

We used Cox regression to compute standardized hazard ratios. The baseline characteristics considered as covariates in Cox regression were sex, age, body mass index, systolic blood pressure and history of cardiovascular disease. We identified covariates to be retained in the analyses by a step-down procedure, removing the least significant covariates at each step until all *P*-values of covariates were less than 0.05. We applied the generalized *R*2 statistic to assess the contribution of HFrEF classifiers to risk over and beyond other risk factors.

## Results

### Characteristics of participants

Clinical characteristics of all 884 study participants comprising HFrEF patients and controls are presented in Table 1. Overall, patients with HFrEF as compared with control individuals were more likely to be older, and to have lower eGFR and hypertension.

### Identification of HFrEF biomarkers

Univariate analysis and correction for multiple testing identified a pattern of 103 distinct HFrEF-related peptide biomarkers which differed significantly (p<0.05) between HFrEF and control proteomic profiles. Overall 65 of the 103 peptides could be characterized by sequence and post-translational modifications (Table 3). The majority of the sequenced peptides originated from constituents of the extracellular matrix (ECM), i.e. fragments of various types of mostly fibrillar collagens. The collagens comprise type I (N = 34), II (N = 3), III (N = 13), V (N = 1), XVI (N = 1) and XXIV (N = 1), respectively. Other peptides originated e.g. from alpha-1-antitrypsin, apolipoprotein A-I, complement C3, fibrinogen beta chain, retinol-binding protein 4, and histone-lysine N-methyltransferase MLL4/WBP7. Comparisons between the HFrEF-related pattern identified in this study and the recently published patterns of 85 and 273 urinary peptides related to preclinical LVDD (classifier HF1) [6,33] and CKD (classifier CKD273) [34] revealed 7 common peptides for the LVDD pattern and 24 common peptides for the CKD pattern (Table 3). For 5 out of the 7 peptides in common with LVDD sequence information was available with 4 originating from type I collagen and 1 from apolipoprotein A-1. These 5 common sequenced peptides also showed a comparable differential excretion in both conditions.The mass spectrometry amplitude data of all peptides of all study subjects is provide as supporting information (S1, S2 and S3 Tables).

**Table 3:** Sequenced peptides constituting the HFrEF-related peptide panel and their differential excretion between HFrEF and Controls.

Peptide ID	Mass	Theor. mass	Sequence	Protein Symbol	Protein name	Accession number	Start AA	Stop AA	HFrEF DE^1^	Com
38879	1439.66	1439.66	TIDEKGTEAAGAMF	SERPINA1	Alpha-1-antitrypsin	P01009	363	376	2.5 ± 1.3	2
40294	1452.66	1452.71	DEPPQSPWDRVK	APOA1	Apolipoprotein A-I	P02647	25	36	2.2 ± 1.5	1
67217	1933.88	1933.89	GDDGEAGKPGRpGERGPpGP	COL1A1	Collagen alpha-1(I) chain	P02452	230	249	2.5 ± 2.1	2
67911	1949.89	1949.88	GDDGEAGKpGRpGERGPPGp	COL1A1	Collagen alpha-1(I) chain	P02452	230	249	3.3 ± 2.6	2
2659	860.36	860.35	DDGEAGKpG	COL1A1	Collagen alpha-1(I) chain	P02452	231	239	-3.9 ± 0.5	
64889	1892.87	1892.86	DDGEAGKPGRpGERGPpGp	COL1A1	Collagen alpha-1(I) chain	P02452	231	249	2.2 ± 2.7	
54688	1684.67	1684.71	EpGSpGENGAPGQmGPR	COL1A1	Collagen alpha-1(I) chain	P02452	288	304	-2.0 ± 0.4	
70024	1997.91	1997.89	NSGEPGApGSKGDTGAKGEpGP	COL1A1	Collagen alpha-1(I) chain	P02452	432	453	4.8 ± 2.1	
70635	2013.91	2013.89	NSGEpGApGSKGDTGAKGEpGP	COL1A1	Collagen alpha-1(I) chain	P02452	432	453	1.7 ± 1.0	
65257	1899.85	1899.84	SGEpGApGSKGDTGAKGEpGP	COL1A1	Collagen alpha-1(I) chain	P02452	433	453	8.2 ± 3.3	
108327	2761.31	2761.34	ERGSPGpAGPKGSpGEAGRpGEAGLpGAKG	COL1A1	Collagen alpha-1(I) chain	P02452	510	539	5.7 ± 4.0	2
2505	858.39	858.38	SpGEAGRpG	COL1A1	Collagen alpha-1(I) chain	P02452	522	530	-2.8 ± 0.7	2
15216	1058.48	1058.46	SpGEAGRpGEA	COL1A1	Collagen alpha-1(I) chain	P02452	522	532	7.5 ± 1.7	
57531	1737.78	1737.78	TGSpGSpGPDGKTGPPGpAG	COL1A1	Collagen alpha-1(I) chain	P02452	541	560	-1.5 ± 0.5	2
24117	1194.55	1194.55	SpGPDGKTGPpGP	COL1A1	Collagen alpha-1(I) chain	P02452	546	558	1.5 ± 0.9	2
28561	1265.59	1265.59	SpGPDGKTGPpGPA	COL1A1	Collagen alpha-1(I) chain	P02452	546	559	-5.4 ± 0.5	1,2
5675	911.43	911.43	DGKTGPpGPA	COL1A1	Collagen alpha-1(I) chain	P02452	550	559	-4.6 ± 0.3	2
14906	1050.48	1050.47	DGRpGPpGPpG	COL1A1	Collagen alpha-1(I) chain	P02452	562	572	-2.7 ± 0.5	2
58941	1765.81	1765.81	GPpGEAGKpGEQGVpGDLG	COL1A1	Collagen alpha-1(I) chain	P02452	650	668	-1.9 ± 0.4	2
21365	1154.51	1154.52	PpGEAGKpGEQG	COL1A1	Collagen alpha-1(I) chain	P02452	651	662	2.1 ± 1.4	2
34724	1366.62	1366.64	pPGEAGKpGEQGVp	COL1A1	Collagen alpha-1(I) chain	P02452	651	664	1.8 ± 0.8	
56139	1708.79	1708.79	pPGEAGKpGEQGVpGDLG	COL1A1	Collagen alpha-1(I) chain	P02452	651	668	-2.2 ± 0.6	
85315	2281.98	2281.98	ANGApGNDGAKGDAGApGApGSQGApG	COL1A1	Collagen alpha-1(I) chain	P02452	699	725	-1.5 ± 0.4	
55582	1697.74	1697.72	NGApGNDGAKGDAGApGApG	COL1A1	Collagen alpha-1(I) chain	P02452	700	719	-1.4 ± 0.3	2
80308	2194.96	2194.95	NGAPGNDGAKGDAGApGApGSQGApG	COL1A1	Collagen alpha-1(I) chain	P02452	700	725	3.5 ± 1.5	
17694	1096.48	1096.48	ApGDRGEpGpP	COL1A1	Collagen alpha-1(I) chain	P02452	798	808	-3.8 ± 0.5	2
32171	1321.59	1321.59	ApGDRGEpGPpGPA	COL1A1	Collagen alpha-1(I) chain	P02452	798	811	-1.6 ± 0.5	1
35339	1378.61	1378.61	ApGDRGEpGPpGPAG	COL1A1	Collagen alpha-1(I) chain	P02452	798	812	-1.4 ± 0.4	1,2
82784	2236.98	2236.98	ADGQpGAkGEpGDAGAKGDAGPpGP	COL1A1	Collagen alpha-1(I) chain	P02452	819	843	2.9 ± 2.0	1
63209	1860.83	1860.82	EGSpGRDGSpGAKGDRGET	COL1A1	Collagen alpha-1(I) chain	P02452	1021	1039	-2.4 ± 1.1	2
70674	2014.90	2014.89	EGSpGRDGSpGAKGDRGETGP	COL1A1	Collagen alpha-1(I) chain	P02452	1021	1041	1.5 ± 0.9	2
61711	1828.85	1828.83	SpGRDGSpGAKGDRGETGP	COL1A1	Collagen alpha-1(I) chain	P02452	1023	1041	6.4 ± 3.0	
44618	1523.74	1523.73	VGPpGPpGPpGpPGPPS	COL1A1	Collagen alpha-1(I) chain	P02452	1177	1193	-1.4 ± 0.5	
50593	1619.79	1619.79	VGPpGPpGPPGPPGPPSAG	COL1A1	Collagen alpha-1(I) chain	P02452	1177	1195	5.1 ± 2.8	
48093	1579.68	1579.73	GpAGPRGERGPpGESGA	COL1A2	Collagen alpha-2(I) chain	P08123	583	599	2.9 ± 3.1	
138279	3596.70	3596.74	EVGKpGERGLHGEFGLpGpAGpRGERGPPGESGAAGP	COL1A2	Collagen alpha-2(I) chain	P08123	566	602	4.4 ± 1.8	
76960	2132.91	2132.98	GARGpEGAQGPRGEpGTPGSpGP	COL2A1	Collagen alpha-1(II) chain	P02458-1	381	403	-2.2 ± 0.6	
15776	1068.45	1068.48	GERGETGPpGP	COL2A1	Collagen alpha-1(II) chain	P02458-1	822	832	-1.5 ± 0.6	
16976	1084.43	1084.43	DGpSGAEGpPGp	COL2A1	Collagen alpha-1(II) chain	P02458-1	962	973	-1.1 ± 0.8	
30699	1299.58	1299.58	DGApGKNGERGGpG	COL3A1	Collagen alpha-1(III) chain	P02461	587	600	-2.4 ± 1.1	
37698	1422.68	1422.67	GLpGTGGPpGENGKPG	COL3A1	Collagen alpha-1(III) chain	P02461	642	657	-2.7 ± 0.5	
38798	1438.67	1438.67	GLpGTGGPpGENGKpG	COL3A1	Collagen alpha-1(III) chain	P02461	642	657	-3.5 ± 0.4	
54525	1680.75	1680.76	GLpGTGGPpGENGKpGEp	COL3A1	Collagen alpha-1(III) chain	P02461	642	659	-1.5 ± 0.4	
61304	1818.83	1818.84	GLpGTGGPpGENGKPGEPGp	COL3A1	Collagen alpha-1(III) chain	P02461	642	661	6.7 ± 5.4	2
61945	1834.82	1834.83	GLpGTGGPpGENGKpGEPGp	COL3A1	Collagen alpha-1(III) chain	P02461	642	661	4.0 ± 5.3	
45950	1551.70	1551.68	GTGGPpGENGKpGEpGP	COL3A1	Collagen alpha-1(III) chain	P02461	645	661	4.7 ± 3.4	
52769	1649.73	1649.78	ApGAPGGKGDAGAPGERGp	COL3A1	Collagen alpha-1(III) chain	P02461	667	685	-1.3 ± 0.7	
36784	1405.69	1405.69	DGVPGKDGPRGPTGP	COL3A1	Collagen alpha-1(III) chain	P02461	752	766	2.4 ± 2.2	
18943	1114.49	1114.49	SpGERGETGPp	COL3A1	Collagen alpha-1(III) chain	P02461	796	806	-3.7 ± 0.4	
95746	2507.13	2507.13	ApGQNGEPGGkGERGAPGEkGEGGPpG	COL3A1	Collagen alpha-1(III) chain	P02461	814	840	7.7 ± 3.0	
71171	2023.91	2023.92	GEPGGkGERGApGEKGEGGpPG	COL3A1	Collagen alpha-1(III) chain	P02461	819	840	3.2 ± 2.3	2
69882	1993.88	1993.88	SEGSPGHpGQPGpPGpPGApGP	COL3A1	Collagen alpha-1(III) chain	P02461	1174	1195	2.7 ± 2.0	
113351	2887.35	2887.37	GpSGpVGpPGLAGERGEQGPpGPTGFQGLPG	COL5A2	Collagen alpha-2(V) chain	P05997	651	681	5.1 ± 5.3	
41770	1473.63	1473.66	GPpGpAGERGHpGApG	COL16A1	Collagen alpha-1(XVI) chain	Q07092-2	1401	1416	-1.5 ± 1.3	
67723	1945.88	1945.77	QGDVGPpGEmGmEGPPGTEG	COL24A1	Collagen alpha-1(XXIV) chain	Q17RW2-2	998	1017	34.2 ± 3.2	
108021	2754.27	2754.28	EGVQKEDIPPADLSDQVPDTESETR	C3	Complement C3 (C3g fragment)	P01024	955	979	-2.2 ± 0.4	
51184	1630.83	1630.83	EEAPSLRPAPPPISGGG	FGB	Fibrinogen beta chain	P02675	54	70	-4.3 ± 0.6	
19046	1116.53	1116.57	PTSRYIHFP	WBP7	Histone-lysine N-methyltransferase	Q9UMN6	2030	2038	13.1 ± 0.8	
18300	1106.50	1106.54	AQYEEIAQR	KRT4	Keratin	F5H8K9	293	301	2.8 ± 1.4	
49958	1608.73	1608.73	SGDSDDDEPPPLPRL	PGRMC1	Membrane associated progesterone receptor component 1	O00264	54	68	-1.8 ± 1.1	2
14071	1032.50	1032.54	RVAPEEHPV	POTEF	POTE ankyrin domain family member F	A5A3E0	795	803	-2.5 ± 0.4	
60751	1807.81	1807.88	SVDETGQmSATAKGRVR	RBP4	Retinol-binding protein 4	P02753	64	80	-1.6 ± 0.6	
53181	1653.88	1653.90	SGSVIDQSRVLNLGPI	UMOD	Uromodulin	P07911	589	604	-1.8 ± 0.8	2
44633	1523.84	1523.87	VIDQSRVLNLGPIT	UMOD	Uromodulin	P07911	592	605	-3.6 ± 0.7	2
20226	1137.67	1137.70	RVLNLGPITR	UMOD	Uromodulin	P07911-2	597	606	52.4 ± 8.5	

Peptides (N = 103) discriminatory for HFrEF. The differential excretion (DE) of peptides between HFrEF and controls has been calculated as follows: For mean MS amplitude HFrEF > mean MS amplitude control: (mean amplitude HFrEF x frequency) / (mean amplitude control x frequency); for mean MS amplitude HFrEF < mean MS amplitude control:—(mean amplitude control x frequency) / (mean amplitude HFrEF x frequency). For calculating means, values from all samples were used, considering 0 for undetected values. HFrEF, Heart failure with reduced ejection fraction; mass, molecular weight in Da; CE time, CE-migration time in min; Peptide ID, polypeptide identifier annotated by the SQL database; Start AA / Stop AA, start and stop amino acid of the identified peptide; Com, peptides commonly shared with the peptide biomarker patterns of the classifier HF1 (1) for preclinical LVDD) [33] and CKD273 (2) for chronic kidney disease; p in peptide sequences, oxidized prolines; m in peptide sequences, oxidized methionines.

### HFrEF classifier modelling

First, the pattern of 103 HFrEF-related peptide biomarkers was used for subsequent SVM based modelling of a proteomic HFrEF disease classifier. The resulting classifier HFrEF103 showed a radial basis function kernel with parameters C = 6.4 and γ = 0.001024. To determine the contribution of the 24 peptides in common with CKD273, we also modelled an HFrEF classifier based on the 79 remaining peptides. The resulting classifier HFrEF79 showed a radial basis function kernel with parameters C = 12.8 and γ = 0.001024. Both classifiers allowed correct classification of all 33 HFrEF patients and all 29 controls of the discovery cohort resulting in sensitivity and specificity of 100% upon complete cross-validation of HFrEF103 derived score factors in ROC analysis.

#### Validation of HFrEF disease classifiers

The discriminatory power of HFrEF103 and HFrEF79 was tested by assessing the proteome profiles of the remaining 94 HFrEF patients with ischemic (N = 34), non-ischemic (N = 45) or uncertain aetiology (N = 15). They were between 38 and 94 years of age with various stages of renal impairment (eGFR between 11 and 140 ml/min/1,73m^2^). The validation data set further comprised profiles of 552 controls without heart failure between 20 and 84 years of age with various stages of renal impairment (eGFR between 44 and 138 ml/min/1,73m^2^).

**Fig 1:**
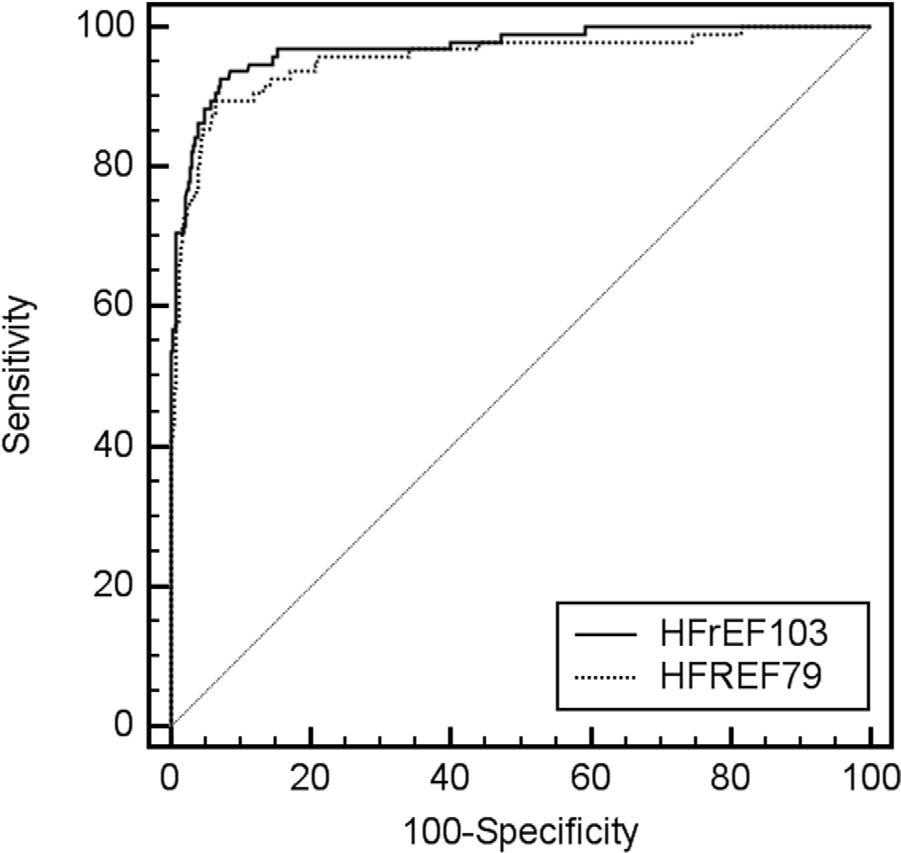
Receiver operating characteristic (ROC) curve for the HFrEF score factors of the validation proteome profile set (N = 646) based on HFrEF103 (solid line) and HFrEF79 (dotted line).

Applying HFrEF103 to this validation cohort resulted in very accurate discrimination between HFrEF patients and control individuals. Sensitivity in patients with HFrEF reached 93.6% (86.6–97.6) and specificity in control individuals with and without impaired kidney function and hypertension 92.9% (90.5–94.9) based on an optimized HFrEF score factor threshold of > -0.083 (Table 4). An HFrEF score factor generated by HFrEF103 above this threshold thus indicated a large increase in the likelihood of HFrEF as specified by a positive likelihood ratio of 13.25. The negative likelihood ratio of 0.07 further indicated that a HFrEF score factor below the threshold largely ruled out HFrEF. An AUC of 0.972 (0.957–0.984; p < 0.0001) in ROC analysis of the score factors also confirmed the discriminatory power of HFrEF103 (Fig 1). A comparable but nonetheless significantly (p = 0.0119) lower discriminatory power of HFrEF79 was shown by an AUC of 0.954 (0.936–0.969; p < 0.0001) (Fig 1) with a sensitivity of 89.5% (81.5–94.8) and a specificity of 93.6% (91.3–95.5) based on an optimized HFrEF score factor threshold of > 0.018.

**Table 4:** Contingency table of HFrEF103 results in the validation cohort and preclinical LVDD evaluation.

	Control	HFrEF	Total	LVDD
**HFrEF classifier positive**	39	88	127	22
**HFrEF classifier negative**	513	6	519	154
**Totals**	552	94	646	176

Classification results of proteome peptide profiles of the validation cohort of 94 HFrEF patients and 552 control individuals without HFrEF as well as the set of 176 LVDD patients by the classifier HFrEF103. HFrEF, Heart failure with reduced ejection fraction.

#### Diagnosis of HFrEF at NYHA class I

In the validation data set 20 (21%) out of the 94 HFrEF patients had no symptoms of heart failure during ordinary activities thus being NYHA class I [35]. These patients can therefore be considered as individuals with preclinical left ventricular systolic dysfunction (LVSD). Importantly, HFrEF103 classified this group with a sensitivity of 95% (75.1–99.9) based on the HFrEF score factor threshold of > -0.083. Further assessing the diagnostic performance for LVSD by using HFrEF103 score factors as a dichotomous variable (0 = HFrEF103 score factor < -0.083; 1 = HFrEF103 score factor > -0.083) in multivariate logistic regression analysis revealed a high stepwise covariate-adjusted (age, sex and eGFR) odds ratio of 650 (37–11353; p < 0.0001).

#### Correlation analysis

Rank correlations (Spearman’s rho) were observed between the HFrEF score factors and age reaching a rho value of ρ = 0.295 (95% CI 0.223–0.364; p < 0.0001) and LVEF reaching a rho value of ρ = -0.359 (95% CI -0.425 to -0.288; p < 0.0001).

### Classification of individuals with preclinical LVDD

HFrEF103 was based on urinary peptide biomarkers relevant for HFrEF and thus primarily systolic dysfunction. This included individuals with preclinical LVSD (NYHA class I). To evaluate if HFrEF103 would also classify individuals with preclinical LVDD as diseased, HFrEF103 was utilized to assess urinary proteome profiles of 176 individuals with preclinical LVDD [6]. If HFrEF103 would classify individuals with LVSD as diseased but not–or at least only to a very limited degree–individuals with LVDD/DLVD, this would suggest considerable differences in pathological mechanisms. The resulting sensitivity in individuals with preclinical LVDD was indeed low and reached only 12.5% (Table 4).

## Discussion

This is a pilot study using CE-MS-based urinary proteomic analysis in HFrEF patients with limited concomitant impairment of kidney function. Major findings include the identification of peptide biomarkers associated with HFrEF and their value for SVM-modelling of the HFrEF disease classifier HFrEF103. This classifier allowed discrimination between HFrEF patients and individuals with LVSD as well as individuals without heart failure with very high sensitivity and specificity, regardless of the aetiology of HFrEF. This opens the possibility of early diagnosis of HFrEF even before the disease progresses to an overt symptomatic stage. Moreover, the observed limited sensitivity in preclinical LVDD opens the possibility of differential heart failure diagnosis.

The remarkable performance of the classifier probably reflects extensive depiction of molecular phenotypic alterations associated with HFrEF. Peptides of fibrillar type I and III collagens were found to be predominantly represented among the identified biomarkers. These collagens are important components of the myocardial extracellular matrix (ECM) [36]. The major component is type I collagen (85% of ECM proteins) which provides cardiac rigidity and determines stiffness [37] while type III collagen (10%) contributes to elasticity [38]. Sustained fibrotic remodelling of the ventricular ECM is part of the molecular pathology of heart failure. Excess deposition of interstitial fibrous tissue, collagen cross-linking increasing resistance to degradation, and altered activities of proteinases involved in ECM turnover and collagen synthesis contribute to remodelling [39,40]. Endomyocardial inflammation propagates those processes [41]. Different combinations of these processes may cause the observed specific patterns of positive and negative differential excretion of peptidic fibrillar collagen fragments. On the functional level, ECM remodelling contributes to perturbed cardiac mechanics together with altered left ventricular chamber geometry and volume [42,43]. While some of the ECM remodelling processes may be characteristic for HFrEF, others appear to be of more common nature as indicated by the peptide biomarker patterns for HFrEF, preclinical LVDD and CKD. These patterns include both, unique as well as common type I and III collagen fragments. Interestingly, the urinary peptide biomarker patterns for HFrEF and preclinical LVDD [33] have only 4 type I collagen fragments in common (Table 3) indicating pronounced differences in ECM remodelling. The fact that the patterns for HFrEF and CKD [34] share 20 fragments of type I and III collagen may be due to the accompanying renal disease as a frequent comorbidity in HFrEF. However, while significant, their relevance for the discriminatory power of HFrEF103 still appears to be rather limited.

In addition to the peptidic collagen fragments, the biomarker pattern includes a peptidic fragment of alpha-1-antitrypsin (AAT), which showed a positive differential excretion (Table 3). Levels of AAT have indeed already been shown to increase progressively across NYHA classes and associate with B-type natriuretic peptide (BNP) [44]. This was suggested to be a compensatory mechanism for the loss of antiprotease activity due to oxidative stress.

In conclusion, in this pilot study HFrEF-related urinary peptide biomarkers identified by CE-MS-based UPA could be utilized to establish a classifier that discriminates between HFrEF patients and controls as well as LVDD patients.

However, there are certain limitations to our study which need consideration. Patients and controls originated from different centres and we did not have a fully independent external validation cohort to assess a potential centre bias. However, the vast majority of patients as well as controls included in the present study were Caucasians from central Europe. Another issue is that peptides were measured in urine only. Therefore we could not determine their source of origin nor could it be established if the changes seen in HFrEF patients are only due to direct cardiac alterations and not also due to non-cardiac organ dysfunction secondary to heart failure. Renal dysfunctions, which are often associated with heart failure [12,13,44] are especially relevant in this context. Therefore HFrEF patients and control individuals included for biomarker discovery have been stratified for mostly no to only mild impairments of kidney function (CKD stage 2) and matched for eGFR to avoid a kidney function bias. Finally, not all identified polypeptides were sequenced.

In spite of these limitations the results are of scientific interest depicting the potential diagnostic power of a multi-biomarker approach mirroring various HFrEF-associated pathological alterations. Large-scale evaluation and validation is needed to assess the full potential value of the UPA-based classifier.

## Supporting Information

S1 Table MS data of HFrEF patients. (TXT)

S2 Table MS data of LVDD patients. (TXT)

S3 Table MS data of Control individuals. (TXT)

## Abbreviations

HFrEF: heart failure with reduced ejection fraction
CE-MS: capillary electrophoresis on-line coupled to electrospray ionization micro time-of-flight mass spectrometry
UPA: urine proteome analysis
AUC: area under the ROC curve
ESI: electrospray ionization
LVDD: preclinical left ventricular diastolic dysfunction
HF: heart failure
LVEF: left ventricular ejection fraction
CKD: chronic kidney disease
NYHA: New York Heart Association
eGFR: estimated glomerular filtration rate
SVM: support vector machine
ROC: receiver operating characteristic
CI: confidence interval
ECM: extracellular matrix
BNP: B-type natriuretic peptide

## References

[1] Brouwers FP, de Boer RA, van der HP, Voors AA, Gansevoort RT, Bakker SJ, et al Incidence and epidemiology of new onset heart failure with preserved vs. reduced ejection fraction in a community-based cohort: 11-year follow-up of PREVEND. Eur Heart J. 2013;34(19): 1424–1431. 10.1093/eurheartj/eht066 23470495

[2] Braunwald E. Biomarkers in heart failure. N Engl J Med. 2008;358(20): 2148–2159. doi: 10.1016/j.ejheart.2008.07.014 pmid: 18480207. 18480207

[3] McMurray JJ, Adamopoulos S, Anker SD, Auricchio A, Bohm M, Dickstein K, et al ESC Guidelines for the diagnosis and treatment of acute and chronic heart failure 2012: The Task Force for the Diagnosis and Treatment of Acute and Chronic Heart Failure 2012 of the European Society of Cardiology. Developed in collaboration with the Heart Failure Association (HFA) of the ESC. Eur Heart J. 2012;33(14): 1787–1847. 10.1093/eurheartj/ehs104 . 22611136

[4] Pouleur AC. Which biomarkers do clinicians need for diagnosis and management of heart failure with reduced ejection fraction? Clin Chim Acta. 2014;443: 9–16. 10.1016/j.cca.2014.10.046 . 25447693

[5] Pejchinovski M, Hrnjez D, Ramirez-Torres A, Bitsika V, Mermelekas G, Vlahou A, et al Capillary zone electrophoresis on-line coupled to mass spectrometry: A perspective application for clinical proteomics. Proteomics Clin Appl. 2015;9(5–6): 453–68. 10.1002/prca.201400113 25641766

[6] Zhang Z, Staessen JA, Thijs L, Gu Y, Liu Y, Jacobs L, et al Left ventricular diastolic function in relation to the urinary proteome: a proof-of-concept study in a general population. Int J Cardiol. 2014;176(1): 158–165. 10.1016/j.ijcard.2014.07.014 . 25065337 PMC4155932

[7] Lemesle G, Maury F, Beseme O, Ovart L, Amouyel P, Lamblin N, et al Multimarker proteomic profiling for the prediction of cardiovascular mortality in patients with chronic heart failure. PLoS One. 2015;10(4): e0119265 10.1371/journal.pone.0119265 . 25905469 PMC4408082

[8] Bosselmann H, Tonder N, Soletormos G, Rossing K, Iversen K, Goetze JP, et al Influence of renal impairment on myocardial function in outpatients with systolic heart failure: an echocardiographic and cardiac biomarker study. Int J Cardiol. 2014;177(3): 942–948. 10.1016/j.ijcard.2014.09.202 . 25449505

[9] Mischak H, Vlahou A, Ioannidis JP. Technical aspects and inter-laboratory variability in native peptide profiling: the CE-MS experience. Clin Biochem. 2013;46(6): 432–443. 10.1016/j.clinbiochem.2012.09.025 . 23041249

[10] Dickstein K, Cohen-Solal A, Filippatos G, McMurray JJ, Ponikowski P, Poole-Wilson PA, et al ESC Guidelines for the diagnosis and treatment of acute and chronic heart failure 2008: the Task Force for the Diagnosis and Treatment of Acute and Chronic Heart Failure 2008 of the European Society of Cardiology. Developed in collaboration with the Heart Failure Association of the ESC (HFA) and endorsed by the European Society of Intensive Care Medicine (ESICM). Eur Heart J. 2008;29(19): 2388–2442. 10.1093/eurheartj/ehn309 . 18799522

[11] Felker GM, Shaw LK, O'Connor CM. A standardized definition of ischemic cardiomyopathy for use in clinical research. J Am Coll Cardiol. 2002;39(2): 210–218. . 11788209 10.1016/s0735-1097(01)01738-7

[12] Mentz RJ, Kelly JP, von Lueder TG, Voors AA, Lam CS, Cowie MR, et al Noncardiac comorbidities in heart failure with reduced versus preserved ejection fraction. J Am Coll Cardiol. 2014;64(21): 2281–2293. 10.1016/j.jacc.2014.08.036 . 25456761 PMC4254505

[13] Braam B, Joles JA, Danishwar AH, Gaillard CA. Cardiorenal syndrome—current understanding and future perspectives. Nat Rev Nephrol. 2014;10(1): 48–55. 10.1038/nrneph.2013.250 . 24247284

[14] Theodorescu D, Wittke S, Ross MM, Walden M, Conaway M, Just I, et al Discovery and validation of new protein biomarkers for urothelial cancer: a prospective analysis. Lancet Oncol. 2006;7(3): 230–240. . 16510332 10.1016/S1470-2045(06)70584-8

[15] Wittke S, Mischak H, Walden M, Kolch W, Radler T, Wiedemann K. Discovery of biomarkers in human urine and cerebrospinal fluid by capillary electrophoresis coupled to mass spectrometry: towards new diagnostic and therapeutic approaches. Electrophoresis. 2005;26(7–8): 1476–1487. . 15765478 10.1002/elps.200410140

[16] Neuhoff N, Kaiser T, Wittke S, Krebs R, Pitt A, Burchard A, et al Mass spectrometry for the detection of differentially expressed proteins: a comparison of surface-enhanced laser desorption/ionization and capillary electrophoresis/mass spectrometry. Rapid Communications in Mass Spectrometry. 2004;18(2): 149–156. . 14745763 10.1002/rcm.1294

[17] Haubitz M, Good DM, Woywodt A, Haller H, Rupprecht H, Theodorescu D, et al Identification and validation of urinary biomarkers for differential diagnosis and dvaluation of therapeutic intervention in ANCA associated vasculitis. Mol Cell Proteomics. 2009;8(10): 2296–2307. 10.1074/mcp.M800529-MCP200 . 19564150 PMC2758757

[18] Jantos-Siwy J, Schiffer E, Brand K, Schumann G, Rossing K, Delles C, et al Quantitative Urinary Proteome Analysis for Biomarker Evaluation in Chronic Kidney Disease. J Proteome Res. 2009;8(1): 268–281. 10.1021/pr800401m 19012428

[19] Dakna M, He Z, Yu WC, Mischak H, Kolch W. Technical, bioinformatical and statistical aspects of liquid chromatography-mass spectrometry (LC-MS) and capillary electrophoresis-mass spectrometry (CE-MS) based clinical proteomics: a critical assessment. J Chromatogr B Analyt Technol Biomed Life Sci. 2009;877(13): 1250–1258. 10.1016/j.jchromb.2008.10.048 . 19010091

[20] Coon JJ, Zürbig P, Dakna M, Dominiczak AF, Decramer S, Fliser D, et al CE-MS analysis of the human urinary proteome for biomarker discovery and disease diagnostics. Proteomics Clin Appl. 2008;2(7–8): 964–973. . 20130789 10.1002/prca.200800024 PMC2815342

[21] Rossing K, Mischak H, Dakna M, Zürbig P, Novak J, Julian BA, et al Urinary proteomics in diabetes and CKD. J Am Soc Nephrol. 2008;19(7): 1283–1290. 10.1681/ASN.2007091025 . 18448586 PMC2440301

[22] Benjamini Y, Hochberg Y. Controlling the false discovery rate: a practical and powerful approach to multiple testing. J Royal Stat Soc B (Methodological). 1995;57: 125–133.

[23] Girolami M, Mischak H, Krebs R. Analysis of complex, multidimensional datasets. Drug Discov Today Technol. 2006;3(1): 13–19. 10.1016/j.ddtec.2006.03.010 . 24980097

[24] R Development Core Team. R: A language and environment for statistical computing Vienna, Austria: R Foundation for Statistical Computing 2008

[25] Bosselmann H, Egstrup M, Rossing K, Gustafsson I, Gustafsson F, Tonder N, et al Prognostic significance of cardiovascular biomarkers and renal dysfunction in outpatients with systolic heart failure: a long term follow-up study. Int J Cardiol. 2013;170(2): 202–207. 10.1016/j.ijcard.2013.10.064 . 24182673

[26] Staessen J, Bulpitt CJ, Fagard R, Joossens JV, Lijnen P, Amery A. Familial aggregation of blood pressure, anthropometric characteristics and urinary excretion of sodium and potassium—a population study in two Belgian towns. J Chronic Dis. 1985;38(5): 397–407. . 3998054 10.1016/0021-9681(85)90135-3

[27] Kuznetsova T, Staessen JA, Kawecka-Jaszcz K, Babeanu S, Casiglia E, Filipovsky J, et al Quality control of the blood pressure phenotype in the European Project on Genes in Hypertension. Blood Press Monit. 2002;7(4): 215–224. . 12198337 10.1097/00126097-200208000-00003

[28] Levey AS, Stevens LA, Schmid CH, Zhang Y, Castro AF III, Feldman HI, et al for the CKD-EPI (Chronic Kidney Disease Epidemiology Collaboration) A new equation to estimate glomerular filtration rate. Ann Intern Med. 2009;150(9): 604–612. . 19414839 10.7326/0003-4819-150-9-200905050-00006 PMC2763564

[29] Girolami M, Mischak H, Krebs R. Analysis of complex, multidimensional datasets. Drug Discov Today Technol. 2006;3(1): 13–19. 10.1016/j.ddtec.2006.03.010 . 24980097

[30] Yang ZR. Biological applications of support vector machines. Brief Bioinform. 2004;5(4): 328–338. . 15606969 10.1093/bib/5.4.328

[31] Decramer S, Wittke S, Mischak H, Zurbig P, Walden M, Bouissou F, et al Predicting the clinical outcome of congenital unilateral ureteropelvic junction obstruction in newborn by urinary proteome analysis. Nat Med. 2006;12(4): 398–400. . 16550189 10.1038/nm1384

[32] Theodorescu D, Fliser D, Wittke S, Mischak H, Krebs R, Walden M, et al Pilot study of capillary electrophoresis coupled to mass spectrometry as a tool to define potential prostate cancer biomarkers in urine. Electrophoresis. 2005;26(14): 2797–2808. . 15981297 10.1002/elps.200400208

[33] Kuznetsova T, Mischak H, Mullen W, Staessen JA. Urinary proteome analysis in hypertensive patients with left ventricular diastolic dysfunction. Eur Heart J. 2012;33(18): 2342–2350. 10.1093/eurheartj/ehs185 . 22789915 PMC3705161

[34] Good DM, Zürbig P, Argiles A, Bauer HW, Behrens G, Coon JJ, et al Naturally occurring human urinary peptides for use in diagnosis of chronic kidney disease. Mol Cell Proteomics. 2010;9(11): 2424–2437. 10.1074/mcp.M110.001917 . 20616184 PMC2984241

[35] Weber KT. Cardiac interstitium in health and disease: the fibrillar collagen network. J Am Coll Cardiol. 1989;13(7): 1637–1652. . 2656824 10.1016/0735-1097(89)90360-4

[36] Brilla CG, Zhou G, Rupp H, Maisch B, Weber KT. Role of angiotensin II and prostaglandin E2 in regulating cardiac fibroblast collagen turnover. Am J Cardiol. 1995; 76(13): 8D–13D. . 7495221 10.1016/s0002-9149(99)80485-8

[37] Weber KT. Extracellular matrix remodeling in heart failure: a role for de novo angiotensin II generation. Circulation. 1997;96(11): 4065–4082. . 9403633 10.1161/01.cir.96.11.4065

[38] Toprak G, Yuksel H, Demirpence O, Islamoglu Y, Evliyaoglu O, Mete N. Fibrosis in heart failure subtypes. Eur Rev Med Pharmacol Sci. 2013;17(17): 2302–2309. . 24065222

[39] Lofsjogard J, Kahan T, Diez J, Lopez B, Gonzalez A, Edner M, et al Biomarkers of collagen type I metabolism are related to B-type natriuretic peptide, left ventricular size, and diastolic function in heart failure. J Cardiovasc Med (Hagerstown). 2014;15(6): 463–469. 10.2459/01.JCM.0000435617.86180.0b . 24983265

[40] Passino C, Barison A, Vergaro G, Gabutti A, Borrelli C, Emdin M, et al Markers of fibrosis, inflammation, and remodeling pathways in heart failure. Clin Chim Acta. 2014;443: 29–38. 10.1016/j.cca.2014.09.006 . 25218738

[41] Barasch E, Gottdiener JS, Aurigemma G, Kitzman DW, Han J, Kop WJ, et al The relationship between serum markers of collagen turnover and cardiovascular outcome in the elderly: the Cardiovascular Health Study. Circ Heart Fail. 2011;4(6): 733–739. 10.1161/CIRCHEARTFAILURE.111.962027 . 21900186 PMC3263368

[42] Barasch E, Gottdiener JS, Aurigemma G, Kitzman DW, Han J, Kop WJ, et al Association between elevated fibrosis markers and heart failure in the elderly: the cardiovascular health study. Circ Heart Fail. 2009;2(4): 303–310. 10.1161/CIRCHEARTFAILURE.108.828343 . 19808353 PMC2993567

[43] Lubrano V, Papa A, Pingitore A, Cocci F. alpha-1 Protein evaluation to stratify heart failure patients. J Cardiovasc Med (Hagerstown). 2014; 10.2459/JCM.0000000000000016 . 24911195

[44] Valente MA, Voors AA, Damman K, van Veldhuisen DJ, Massie BM, O'Connor CM, et al Diuretic response in acute heart failure: clinical characteristics and prognostic significance. Eur Heart J. 2014;35(19): 1284–1293. 10.1093/eurheartj/ehu065 . 24585267

